# Laparoscopic Reversal of Tubal Sterilization; A Retrospective Study Over 135 Cases

**DOI:** 10.3389/fsurg.2018.00079

**Published:** 2019-01-09

**Authors:** Pierre Arnaud Godin, Konstantinos Syrios, Gwennaelle Rege, Sami Demir, Efstratia Charitidou, Olivier Wery

**Affiliations:** ^1^Department of Obstetrics and Gynecology, St-Vincent Clinic, Rocourt, Belgium; ^2^Department of Obstetrics and Gynecology, Mitera Hospital, Athens, Greece; ^3^Department of Mathematics, National Technical University of Athens, Athens, Greece

**Keywords:** tubal sterilization, reversal, reanastomosis, laparoscopy, robotically assisted

## Abstract

**Objectives:** To evaluate the pregnancy and delivery rates of laparoscopic tubal reanastomosis.

**Study Design:** From 2003 to 2013, 135 laparoscopic tubal reversals were performed according to the four stitch technique. The parameters studied, included positive pregnancy test, miscarriage, ectopic pregnancy, termination of pregnancy, term delivery, post-operative time to conception, post-operative hysterosalpingography, and spermogram.

**Results:** From the 135 patients operated, 93 fulfilled the inclusion criteria. The age of patients varied from 27 to 47 years old. All ages combined, positive β-HCG blood sample rate was 75.3% (95% CI: 65.0–83.4%) and term delivery 52.7% (95%CI: 42.1–3.0%). The age-adjusted pregnancy and delivery rates were as follows:

27–35 y.o. (*n* = 23) 95.7% (95%CI: 76.0–99.8%) and 73.9% (95%CI: 51.3–88.9%),
36–39 y.o. (*n* = 40) 77.5% (95%CI: 61.1–88.6%) and 47.5% (95%CI: 31.8–63.7%),
40–42 y.o. (*n* = 19) 68.4% (95%CI: 43.5–86.4%) and 52.6% (95%CI: 29.5–74.8%),
43–47 y.o. (*n* = 11) 36.4% (95%CI: 12.4–68.4%) and 27.3% (95%CI: 7.3–60.7%).

**Conclusions:** In our series the pregnancy and delivery rates after laparoscopic reversal of tubal sterilization is estimated at 75.3 and 52.7%, respectively. For women with tubal sterilization and no other infertility factors, reanastomosis can restore anterior natural fertility related to age. Laparoscopic reversal should be proposed systematically to patients and performed by well-trained laparoscopists, avoiding potentially the inconvenient and adverse outcomes of an IVF treatment. Although, it may seem a more cost-effective technique compared to robotically assisted reversal, a prospective randomized trial could answer this question.

## Introduction

Tubal sterilization is a commonly spread method of contraception worldwide. Its use largely varies from one country to another, depending on socio-economic level, religion and medical facilities. For example, according to the World Health Organization (WHO), tubal sterilization in Belgium is estimated at 8.4%, in Greece 5.8%, in China 28.7% and reaches even 47.4% in Dominican Republic (1).

Despite the definitive result of this procedure, many women express regret. Up to 14.3% or even 30% has been reported to ask for tubal reversal, with finally only 1.1% of women sterilized being operated (2). Change of mind is usually due to change of marital status, loss of a child or change of attitude (3). An important risk indicator for regret of sterilization is young age (4).

The options for patients are either IVF or surgical tubal anastomosis. For a long time the standard of choice for surgical treatment has been through a mini laparotomy using microsurgical techniques. Laparoscopy has been introduced with increasingly good outcome and recently robot-assisted laparoscopy has shown promising results.

The aim of this retrospective study is to present our experience in pure laparoscopic tubal reversal in terms of pregnancy and delivery rates and to demonstrate that in expert hands this procedure is an alternative option in the era of IVF and ultra-sophisticated robot technology.

## Materials and Methods

### Patients

Our study includes patients who consulted our Center of Reproductive Medicine, St-Vincent Clinic, Rocourt, Belgium, asking for reversal after tubal sterilization. Patients were informed for the different options of treatment, IVF vs. surgery and opted for tubal surgery. The recruitment period was from June 2003 to September 2013. The study protocol was approved by the Ethics Committee of St-Vincent Clinic, Rocourt, Belgium, President: Dr Michel Masson. All subjects gave written informed consent in accordance with the Declaration of Helsinki. The raw data supporting the conclusions of this manuscript will be made available by the authors, without undue reservation, to any qualified researcher.

St-Vincent Rocourt is a tertiary reference center for medical and surgical reproductive medicine in Wallonia, Belgium. The patients were referred by their general practitioners, their gynecologists or sought consultancy on their own. All patients to whom reversal was proposed had no other infertility factors. All surgeries were performed by the same surgeon, P. A. G., according to the four stitch technique. Among the 135 patients operated, 28 were out of contact, 13 were associated with an abnormal spermogram and were therefore excluded from the study, and 1 patient was diagnosed for ovary cancer 9 months after the tubal reanastomosis and was also excluded. The final sample under study consisted of 93 patients. All pregnancies included in the pregnancy rates, were defined as positive serum hCG result and were achieved spontaneously, without any medically assisted procedure (e.g., intrauterine insemination or IVF). No surgical adverse outcomes were encountered. Post-operative hysterosalpingography was prescribed at 3 months interval and success was defined as at least one permeable tube.

### Surgical Procedure

Laparoscopic tubal anastomosis applies the conventional microsurgery principles used in laparotomy. Before starting the laparoscopy, a hysteroscopy was systematically performed to rule out any cavity deformities. A uterine manipulator with dye test was then placed. Insufflation at the level of the umbilicus was made with a Veress needle and a 10 mm trocar was introduced. After inspecting the pelvic cavity, we concluded as to the feasibility of the procedure. If pelvic adhesions were too dense and/or the destruction of tubes was too expanded due to endometriosis or to previous method of sterilization, the procedure was stopped.

The preferred technique in our center is the four stitch technique using polypropylene sutures. Three more 5 mm trocars were introduced in a straight line two fingers above the pubic symphysis and 3 mm laparoscopic instruments were introduced. At first, the scar tissue and serosa of the proximal tubal stump were removed using a fine forceps and a unipolar ovarian drilling needle in cut mode, 10 watts. No hemostatic infiltration was used, e.g., vasopressin or epinephrine. Hemostasis was achieved each time very carefully and electively to the bleeding vessel usually using monopolar coagulation. The proximal stump was then cut transversely with cold scissors. Tubal patency was determined by lavage of methylene blue infused through the uterine manipulator. The distal stump was treated the same way and tubal patency was determined using a tubal catheter. A fimbrioplasty was performed when necessary.

If the gap was too important and the tension too high at the level of the mesosalpinx, one or two 5–0 polypropylene intracorporeal stitches were placed, so as to approximate the proximal and distal stumps and reduce tissue tension.

The muscle layer of the two stumps was then sutured with a 7–0 or 8–0 polypropylene suture with four throws depending on the size of the lumen and starting at the 6 o'clock site. In order to align the mucosa, care was taken to follow an outer to inner and an inner to outer direction of the suture so as to place the knot outside the lumen. If possible, attempts were made not to transfix the mucosa. Then followed the 12 o'clock site and finally the 3 and 9 o'clock sites. Variants were possible in the order of sutures depending on anatomical difficulties of each case, but the most constant step was the 6 o'clock suture placed first.

After the anastomosis of the muscle layer was completed, tubal patency was checked with visualization of methylene blue flow through the fimbriae. A tubal splint for the suture of the muscular layer was rarely used, although it may be useful when alignment of the lumen seemed difficult or the tubal stumps were discongruent. Finally, the serosal layer was approximated with two or three 5–0 polypropylene sutures. Experience shows that when the surgeon operates from the left side of the patient, the right tube is easier to repair, taking less time.

### Statistical Analyses

All statistical analyses were performed using R Statistical Software, version 3.15.1 (The R Foundation for Statistical Computing, Vienna, Austria). The level of statistical significance was set at 5%. Shapiro-Wilk normality tests were carried out and indicated that age at surgery and sperm mobility, as measured by spermograms, are normally distributed quantitative variables (*p*-values > 0.05) contrary to sperm concentration (*p*-value = 0.003). In the following sections, qualitative parameters are presented by their absolute (N) and relative (%) frequencies, whereas quantitative characteristics are presented by their mean and standard deviation (SD), or by their median and interquartile range (IQR) when severely skewed according to the normality-test results.

We were primarily interested in two outcome variables; namely the pregnancy rate and the delivery rate in the sample. At a second level, we were also interested in the time from laparoscopic operation up to first pregnancy. Note that successful delivery refers to the first birth given by a patient in a series of potentially more than one deliveries during the follow-up period. What is more, time to first pregnancy was recorded as one of the following three categories: <6 months, more than 6 months but less than a year, more than a year.

For properly summarizing rates, the sample estimates and the respective 95% confidence intervals (95% CI) for the underlying (“true”) proportions have been calculated based on the one-sample proportion test with Yate's continuity correction. The related *X*^2^ statistic values of the proportion tests are also given along with the related degrees of freedom (df); the null hypothesis tested is that the true rate equals 50% vs. the alternative hypothesis that it is different than 50%. For the investigation of the potential association between pregnancy or delivery and a qualitative factor, such as age group, Fisher's exact test was employed. Note that the variable of age was categorized into four levels: <36 years old, 36–39 years old, 40–42 years old and more than 42 years old, following the Belgian register for assisted procreation (BELRAP). Finally, Cox's proportional hazards model was implemented to check on the potential association between time to pregnancy and one of the following factors: Age group, sterilization type, post-operative HSG and spermogram. Due to the categorical nature of the latter covariates of interest (reaching up to four levels/categories each), combined with the total sample size of the study, only univariate analyses were performed in order to maintain sufficient statistical power under the general rule of thumb of at least 20 observations per level of each categorical variable. The corresponding survival analysis results include hazard ratios, 95% confidence intervals and log-rank test statistics.

## Results

The age at surgery of the 93 non-excluded patients ranged from 27 to 47 years with mean age equal to 37.5 years (*SD* = 4.1). The various age groups are distributed as follows: there were 23 women (24.7%) of age <36 years old, 40 women (43.0%) of age between 36 and 39 years old, 19 women (20.4%) of age between 40 and 42 years old and finally 11 women (11.8%) of age above or equal to 43 years old.

Concerning the spermogram results of the women's male partners, there were 73 normal spermograms (78.5%), 6 spermograms of intermediate type (6.4%), 4 (4.3%) were identified as pathological but had previously a child with spontaneous conception and there are also 10 (10.8%) women with no spermogram results in their corresponding medical record. Spermogram normality was defined by the standard WHO classification and intermediate type when one pathological parameter was found. It is interesting that regarding the intermediate type spermograms, 4 of the corresponding women did not get pregnant in the follow-up period, while the remaining 6 women did get pregnant with only 2 of them actually delivering. The three parameters related to the spermogram results in the sample are summarized as follows: the median concentration was 70.0 million/ml (IQR = 83.0 million/ml), the mean sperm mobility was 55.9% (*SD* = 11.4%) and the median morphology value was 7.0% (IQR = 6.5%). On the other hand, the post-operative hysterosalpingography (*HSG*) was successful in 40 cases (43.0%), failed in 6 cases (6.5%), and was absent for the remaining 47 women (50.5% of the sample). Four sterilization methods were reported: 39 clips (41.9%), 40 coagulation/sections (43.0%), 7 Pomeroy techniques (7.5%), and 7 rings (7.5%).

There were 13 women that did get pregnant spontaneously after surgery, but did not deliver, and were later lost to follow up; So, it is unknown to us whether they proceeded to IVF or not. Out of 13 women in total (14% of the sample) who certainly proceeded to IVF (2 that did get pregnant spontaneously after surgery but did not deliver and 11 that never got pregnant spontaneously after surgery), 6 (46.2%) got pregnant and also delivered successfully.The pregnancy rate in the sample was 75.3% (95%CI, 65.0–83.4%; one-sample proportion test *X*^2^ = 22.75, df = 1, *p*-value < 0.001), while the sample delivery rate was 52.7% (95% CI, 42.1–63.0%; one-sample proportion test *X*^2^ = 0.17, df = 1, *p*-value = 0.67). Among the 70 pregnancies, 2 (2.9%) were interrupted due to medical termination of pregnancy, and also 6 (8.6%) were of ectopic nature (EP). Finally, regarding miscarriage rates among pregnant women, there were 22 positive cases (31.4% of the pregnant patients) while 2 women (2.9%) were still pregnant at the end of the study.

**Figure 1:**
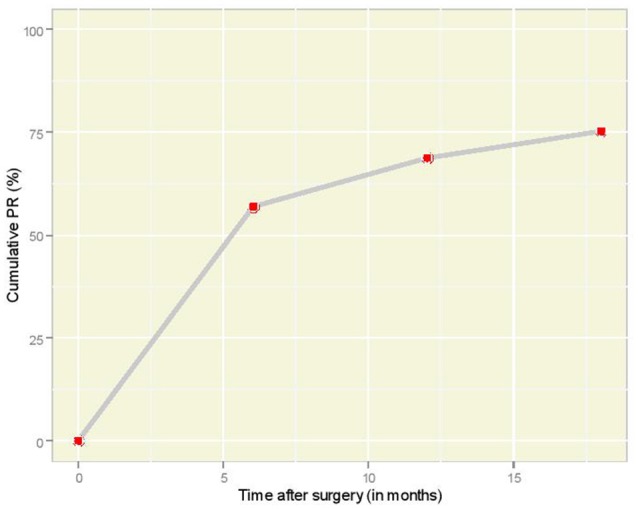
Overall cumulative PR plot according to time interval (in months) after laparoscopic anastomosis.

The cumulative incidence of pregnancy according to time is illustrated in Figure 1. It is interesting that 53 women (57.0%) became pregnant within the first 6 months after the laparoscopic tubal reversal operation. As time went by, the increments of cumulative incidence seemed to drop and one would expect the plot to reach to a plateau, were the follow-up period adequately prolonged.

**Figure 2:**
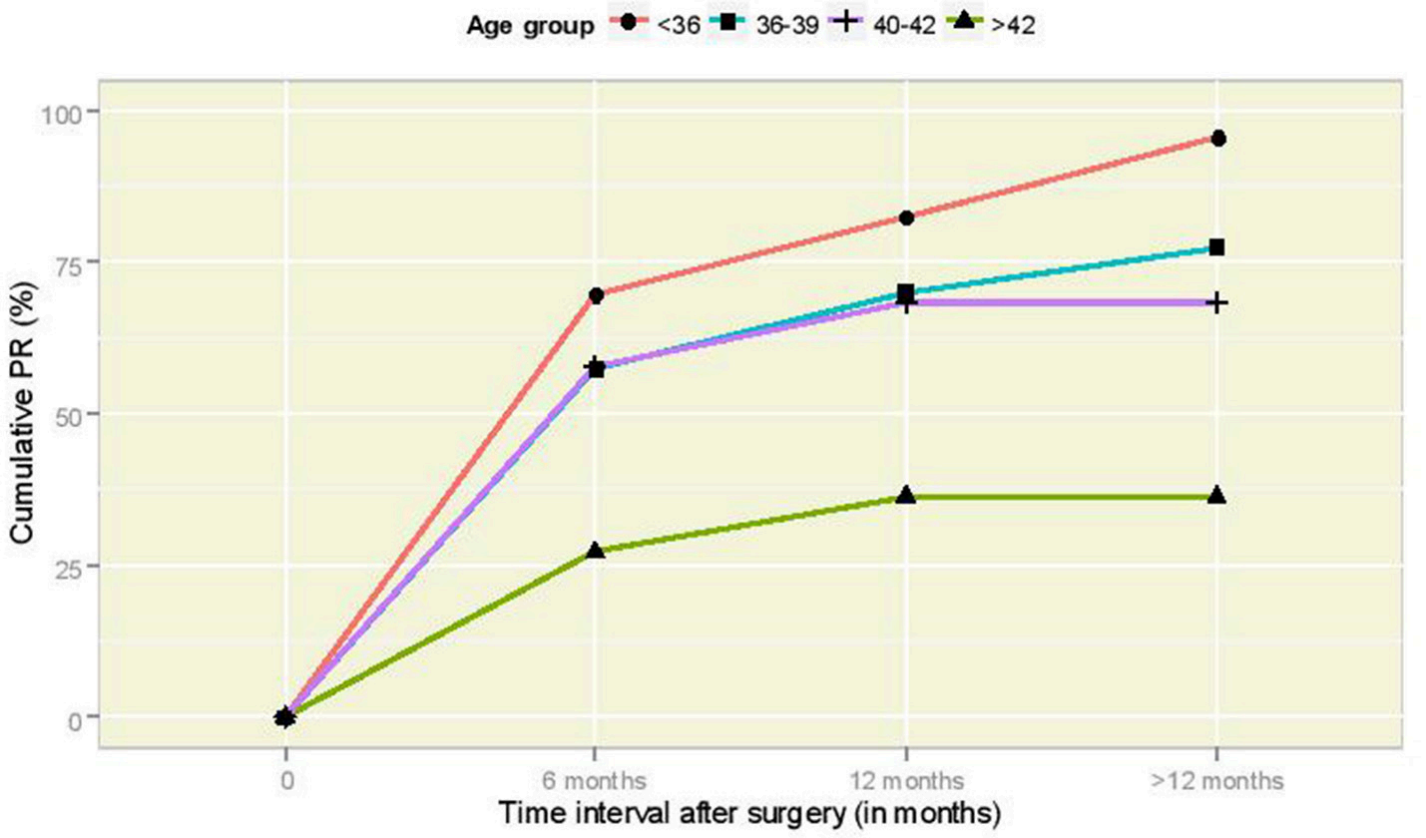
Cumulative PR curves according to time interval (in months) after laparoscopic anastomosis for the different age groups.

Table 1 illustrates the pregnancy and delivery rates after the end of the follow-up period for various factors of primary interest for this study, along with 95% CI figures for the true underlying proportions. The pregnancy rate (PR) was found to vary significantly according to the age at surgery (Fisher's exact test *p*-value = 0.002). The cumulative incidence of pregnancy according to time per age group is illustrated in Figure 2; it is interesting to observe that women of 36–39 years old and 40–42 years old follow very similar paths. The association between sterilization type and PR was not statistically significant (Fisher's exact test *p*-value = 0.58). The relation between PR and post-operative HSG result was statistically significant (Fisher's exact test *p*-value = 0.004) and so was the corresponding relation of PR with the spermogram result (Fisher's exact test *p*-value = 0.04). The successful delivery rate (DR) varied according to the age at surgery in a barely significant fashion (Fisher's exact test *p*-value = 0.057). The association of DR with sterilization type was clearly non-significant (Fisher's exact test *p*-value = 0.29), whereas statistical significance was revealed in the relation between DR and post-operative HSG (Fisher's exact test *p*-value = 0.005). Moreover, DR was statistically significantly associated with the spermogram type (Fisher's exact test *p*-value = 0.003). Note that post-operative HSG was significantly associated with the type of sterilization (Fisher's exact test *p*-value = 0.03).

**Table 1:** Pregnancy (PR) and delivery (DR) rates (%) with respective 95% confidence intervals (95% CI, based on one-sample proportion test for the true underlying rates) at the end of the follow-up period according to various factors of interest.

**Parameter**	**PR**			**DR**		
	***N***	**(%)**	**95% CI**	***N***	**(%)**	**95% CI**
**AGE GROUP**						
<36 y.o.	22	95.7	76.0–99.8%	17	73.9	51.3–88.9%
36-39 y.o.	31	77.5	61.1–88.6%	19	47.5	31.8–63.7%
40-42 y.o	13	68.4	43.5–86.4%	10	52.6	29.5–74.8%
>42 y.o	4	36.4	12.4–68.4%	3	27.3	7.3–60.7%
**STERILIZATION TYPE**						
Clips	29	74.4	57.6–86.4%	23	59.0	42.2–74.0%
Coagulation/section	32	80.0	63.9–90.4%	22	55.0	38.7–70.4%
Pomeroy technique	5	71.4	30.3–94.9%	2	28.6	5.1–69.7%
Ring	4	57.1	20.2–88.2%	2	28.6	5.1–69.7%
**POST-OPERATIVE HSG**						
Unknown	42	89.4	76.1–96.0%	32	68.1	52.9–80.9%
Successful	24	60.0	43.4–74.7%	16	40.0	24.9–56.7%
Failed	4	66.7	24.1–94.0%	1	16.7	0.4–64.1%
**SPERMOGRAM**						
Unknown	10	100.0	65.5–100.0%	10	100.0	69.2–100.0%
Normal	50	68.5	56.4–78.6%	35	47.9	36.1–60.0%
Intermediate	6	100.0	51.7–100.0%	2	33.3	4.3–77.7%
Pathological	4	100.0	39.6–100.0%	2	50.0	6.8–93.2%

*The absolute frequency (N) of pregnancies and deliveries per category is also reported*.

As to the univariate results of the Cox proportional hazards models, they are summarized in Table 2. Contrary to sterilization type, there were significant effects of the rest of the factors examined, i.e., age group, post-operative HSG result and spermogram result, on the time until the first pregnancy. The hazard of pregnancy was reduced by 79% for women of over 42 years old compared to the youngest age group (<36 years old). Moreover, the hazard of pregnancy reduced by 62% for women with successful post-operative HSG relative to women with absent HSG results. Also, it was found that normal spermograms are associated with 57% decreased hazard of pregnancy compared to cases with absent spermogram results.

**Table 2:** Univariate Cox proportional hazards analysis for time to pregnancy including hazard ratio (HR), 95% confidence interval (95% CI) and significance (p-value), as well as the results of the associated log-rank test.

**Parameter**	**HR**	**95% CI**	***p*-value**	**Log-rank test**	
				**X^**2**^ (df)**	***p*-value**
Age group				11.7 (3)	0.008
<36 y.o.^a^	–	–	–		
36-39 y.o.	0.66	0.38–1.15	0.13		
40-42 y.o	0.57	0.28–1.14	0.11		
>42 y.o	0.21	0.07–0.62	0.005		
Sterilization type				2.2 (3)	0.53
Clips^a^	–	–	–		
Coagulation/section	0.82	0.31–2.12	0.68		
Pomeroy technique	1.27	0.77–2.11	0.33		
Ring	0.68	0.23–1.92	0.46		
Post-operative HSG				14.1 (2)	<0.001
Unknown^a^	–	–	–		
Successful	0.38	0.23–0.64	<0.001		
Failed	0.59	0.21–1.65	0.32		
Spermogram				9.7 (3)	0.02
Unknown^a^	–	–	–		
Normal	0.43	0.21–0.86	0.01		
Intermediate	1.11	0.40–3.06	0.83		
Pathological	0.68	0.21–2.00	0.52		

a *Reference category*.

## Discussion

The first tubal anastomosis was described by Garcia (5). Since then, many teams have published with quite different results, first using the mini-laparotomy technique and later using the same principles laparoscopically. The first attempt in laparoscopy was made by Sedbon et al. with use of biologic glue in 1989 (6) and the first robot assisted tubal reversal was performed by Falcone et al. (7). Robot gains ground lately, but cost still remains a major drawback.

Many papers exist in the literature of tubal reversal using the mini-laparotomy with pregnancy rates (PRs) varying from 54 to 91%. For example Kim et al. in 1997 reported a 91.6% PR (*n* = 387) (8), Kim et al. also in 1997 54.8% (*n* = 1118) (9), Cha et al. in 2001, 80% (*n* = 44) (10), and Moon et al. in 2012, 85.1% (*n* = 961) (11).

There are no randomized controlled trials comparing the different routes of surgical reversal of tubal sterilization (12). Such studies are difficult to conduct because of the relative rarity of this indication, the technical facilities of the institutions, the differences in costs, the surgeon's skills or even the patient's desires. Most of the studies in the literature are retrospective with different methodologies and with pregnancy rates that vary widely. Definitions are not uniform making comparison difficult, e.g., pregnancy rates can be defined as percentage of positive B-HCG blood test or percentage of on-going pregnancy more than 12 weeks of gestation. Nevertheless, mini laparotomy seems to be comparable with laparoscopy regarding PRs (54–88% vs. 31–85%, (13, 14) and different laparoscopic techniques have been developed to simplify the procedure (4-stitch, 3- stitch, 2-stitch, 1-stitch, fibrin glue, staples, combination of all above).

Concerning the robotic procedure, despite its increased range of indications, no randomized control studies are available. The place of robotic surgery in the management of infertility remains undetermined (15). The best published study to our knowledge is that of Caillet et al. (16), with 97 patients enrolled and a pregnancy rate of 71% all ages combined. The advantages of robotic surgery are well-known, direct passage from laparotomy to robot assisted, learning curve much more easier comparing to laparoscopy, absence of physiologic tremor, comfort for the surgeon, whereas increased operative times and equipment costs are counterbalanced by decreased hospitalization and recovery times compared to open surgery. But as we show in our series, we can have similar results (PR: 75.3% overall) in classic laparoscopy with less cost and possibly similar operative time even though, procedure time was not systematically recorded in our series (mean time: 90–120 min for bilateral reversal). Recently, Van De Water et al. in 2015 published a good series of 88 patients in favor of laparoscopic reversal with similar results to ours (PR: 73% for women < 40 y.o.) (17).

Many factors seem to affect the PRs in tubal reversal with age playing, as expected, a major role. Tubal reversal restores woman's natural fertility; consequently, success rates are inversely proportional to age. Thirty seven years seems to be a pivotal age. According to Boeckxstaens et al. (2), cumulative pregnancy rates are higher for tubal reversal in patients below 37 years old and higher for IVF in patients over 37, even though the comparison did not reach statistical difference (2). Trimbos in 1990 reported a PR of 45% in women between 40 and 45 years old (18) and Petrucco et al. in 2007 a 40% live birth for patients over 40 years old after TR (19). In our study PRs and DRs for women between 40 and 42 y.o. were 68.4 and 52.6% respectively and for women >42 y.o. 36.4 and 27.3% respectively. These results seem to be consistent to the literature and we may explain the high percentages comparing to IVF with the fact that these women do not have other infertility factors besides prior mechanical tubal ligation (e.g., endometriosis). We therefore believe that tubal reversal is an option for patients over 40 if no other infertility factors exist.

The method of previous sterilization is also important, with clips or rings (Filshie, Hulka-Clemens, Yoon) being associated with better results after tubal reversal, comparing to coagulation/section techniques (electrocautery, Pomeroy modified, Parkland), probably due to the lesser tissue destruction. This is not a constant finding in the literature (4, 20–22) and we did not find any such correlation in our sample.

Postoperative tubal length remains also controversial. A cut off of ≥4 cm of tubal length is often suggested to have better results (23–26). Other studies find no such correlation (4, 9, 20, 21). The site of the anastomosis has also been implicated. Isthmus to isthmus anastomosis seems to be associated with better results (22, 26) but this is not a universal finding (8, 9, 20, 26). However, there is concern regarding increased ectopic pregnancy rate, when isthmus to ampulla anastomosis is performed (20). We did not study these parameters. Other factors with inconstant findings are time interval from previous sterilization, BMI, smoking or alcohol intake (27).

Another frequent dilemma is the choice between tubal reanastomosis and IVF treatment. There are no randomized controlled trials, to our knowledge comparing these two methods. According to the American Society for Reproductive Medicine committee opinion in 2015, the results of tubal surgery and IVF are not directly comparable because surgical success is reported as pregnancy rates per patient, whereas IVF success rates are per cycle. Tubal anastomosis has a significantly higher cumulative pregnancy rate than IVF and is more cost effective even without considering costs associated with multiple births (22, 28). According to Messinger et al. in US clinics, tubal reanastomosis seems to be more cost-effective for patients <41 years old, while IVF appears most cost-effective in patients over 41 years old (29).

The advantages of tubal surgery are that it is a one-time, usually minimally invasive, outpatient procedure and patients may attempt conception every month without further intervention and may conceive more than once (28). Adverse outcomes of IVF treatment can be alleviated (e.g., risk of multiple pregnancy, ovarian hyperstimulation syndrome) with the price of slightly increased EP rate [TR: 2–10% vs. IVF: 2%, (28)]. In our series, ectopic pregnancy is estimated at 8.6% among pregnant women. Once more, comparison remains difficult between different series, because of the different operative techniques of the teams. The site of the anastomosis, the number of stitches used, biological glue, staples or even the use of a tubal splint to facilitate the surgical procedure, are factors that may affect tubal patency and consequently influence the ectopic pregnancy rates.

Biases in our study include its retrospective nature, the fact that we have no control for the degree to which patients were actively pursuing conception and the high number of lost follow up patients despite repeated phone calls (*n* = 28). Some patients, not included in the study, were offered tubal reanastomosis because of their denial to an IVF treatment, due to abnormal semen analysis (*n* = 13).

## Conclusions

Laparoscopic tubal reversal is a well-established, minimally invasive procedure in fertility surgery. It is shown in our study that such procedures are feasible when performed by advanced laparoscopists and have good results (75.3% in terms of pregnancy rates, 52.7% in terms of delivery rates). Pregnancy rates, as expected, are inversely proportional to age, leaving place even for patients over 40 years old. The decision regarding whether to perform tubal anastomosis or IVF treatment is left up to the patient after reviewing the pros and cons of each treatment. Randomized controlled trials, although difficult to conduct because of the rarity of the indication, comparing pure laparoscopic vs. robot assisted tubal reversal, could evaluate the patient's benefit in terms of success rates.

## Author Contributions

PG: principal surgeon, project development; KS: associate surgeon, data management, manuscript writing and editing; GR and SD: data collection; EC: statistical analysis; OW: supervisor, head of department.

### Conflict of Interest Statement

The authors declare that the research was conducted in the absence of any commercial or financial relationships that could be construed as a potential conflict of interest.
